# Supplementary material for: LRP2 contributes to planar cell polarity-dependent coordination of motile cilia function
Source: Cell Tissue Res. 2023 Feb 11;392(2):535–51. doi: 10.1007/s00441-023-03757-7 (PMC10172251; doi:10.1007/s00441-023-03757-7)
Supplement: Supplementary file 1 — Supplementary file1 (PDF 6103 KB) [file 441_2023_3757_MOESM1_ESM.pdf]

SUPPLEMENTARY INFORMATION

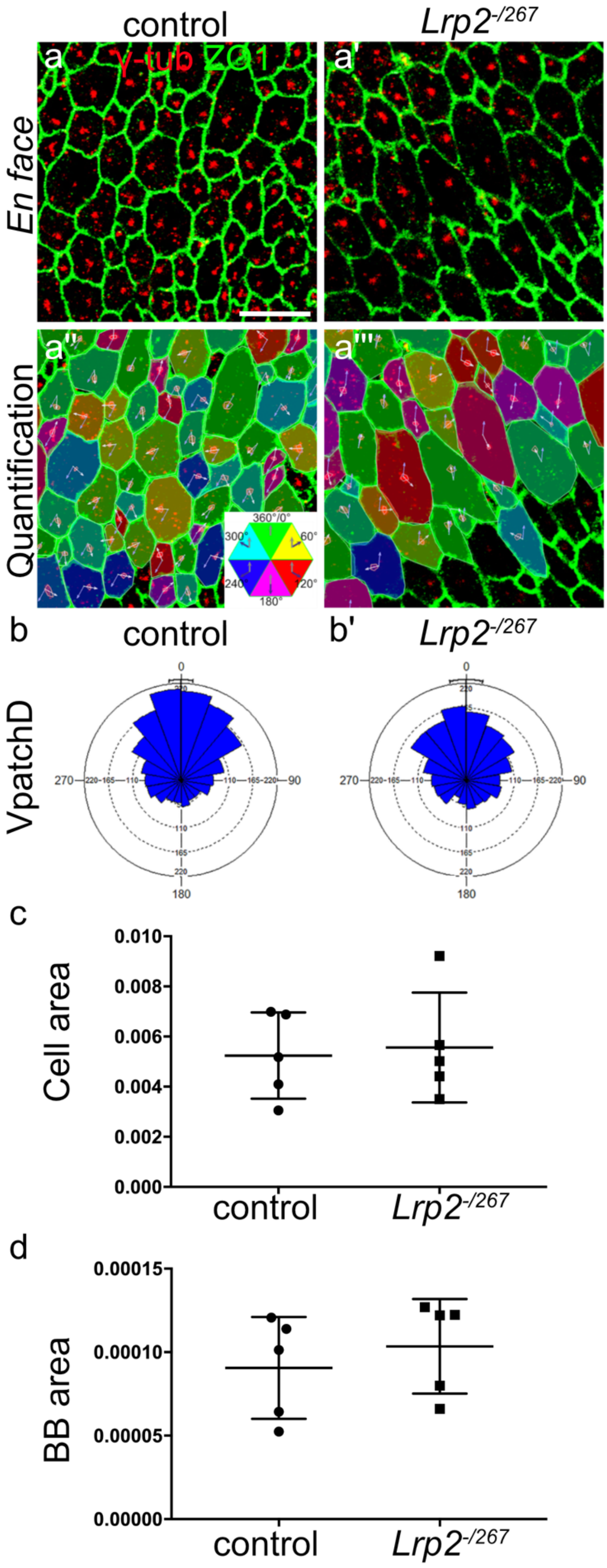

Supplementary Figure 1: Disturbed translational polarity in LRP2-deficient ependymal radial progenitors

(**a- a'**) Panels depict immunodetections of basal body marker γ-tubulin (red) and ZO1 (green) marking primary cilia and apical cell boundaries on *en face* preparations of the lateral wall of newborn control and *Lrp2*$^{-/267}$ mice. Scale bar: 10 µm. (**a''-a'''**) Panels show the same images as in a-a' but with visualization of tissue-wide translational polarity based on displacement of the primary cilium from cell center using the Biotool1 software (github.com/pol51/biotool1). White arrows represent vectors of basal body displacement while blue arrows indicate the mean vectors of displacement across the tissue. Cells with misaligned primary cilium displacement compared to the mean across the tissue are color-coded according to the given scheme. (**b-b'**) Graphical representation of circular statistical analysis using Watson U^2 test (control: 1872 cells, *Lrp2*$^{-/267}$: 1741 cells, 5 animals per genotype) documenting a significantly broader distribution of VpatchD angles around the mean in *Lrp2*$^{-/267}$ ependymal cells as compared with control cells ($p<0.001$). (**c-d**) Using Biotool1 software and analyzing 5 animals per genotype (1872 cells in control and 1741 cells in *Lrp2*$^{-/267}$ tissues), no significant changes were detected in cell surface area (cell area, c) or BB size (BB area, d) comparing genotypes.

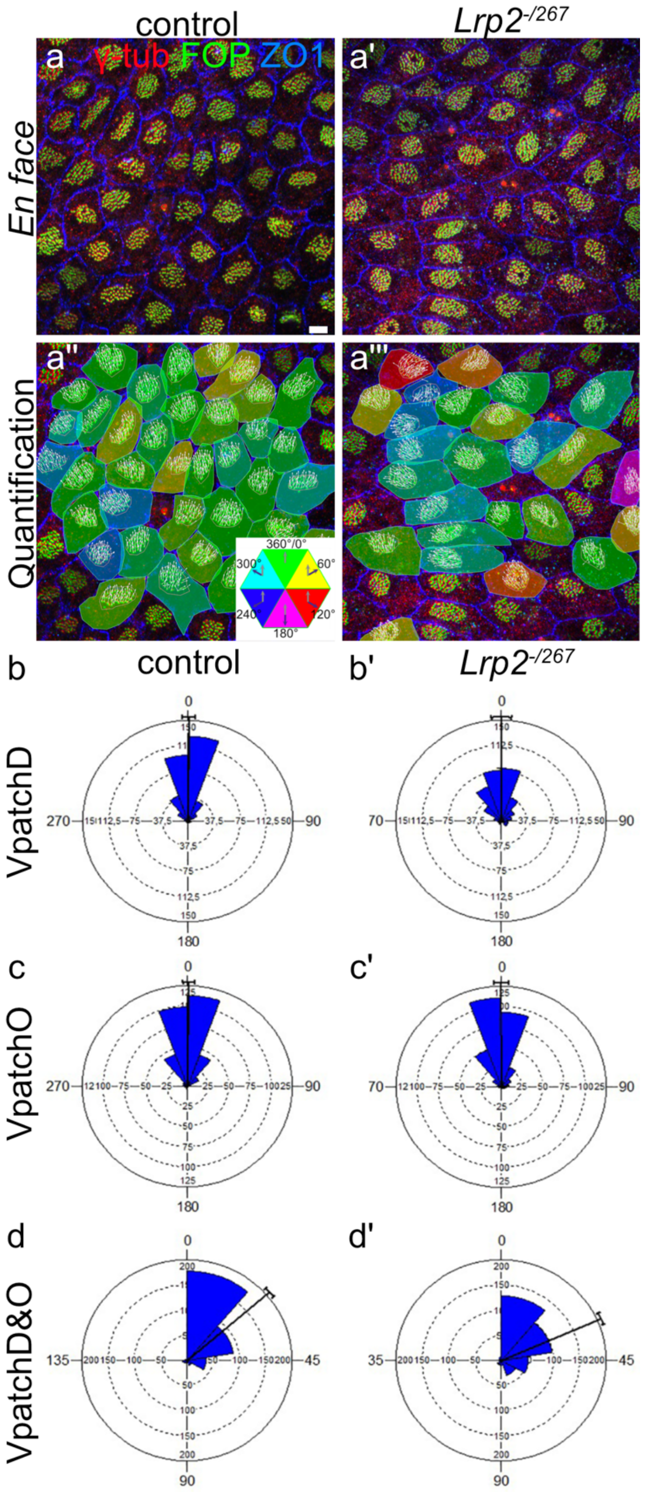

Supplementary Figure 2: Misalignment of ciliary patch displacement and beating orientation in the LRP2-deficient adult ependyma

(a-a') *En face* views of the ventricular lateral wall of adult control and *Lrp2*$^{-/267}$ mice (postnatal day >70) immuno-stained for basal body markers FOP (green) and γ-tubulin (red), as well as for apical cell surface marker ZO1 (blue). Scale bar: 5 µm. **(a''-a''')** Panels depict

the same images as in a-a**'** but visualizing analysis of tissue-wide planar cell polarity using the Biotool1 software. Arrows indicate the vectors from FOP to γ-tubulin immunosignals in individual cilia, defined as Vcil. Color coding of individual cells describes the coordination of ciliary patch displacement. VpatchD was defined from the center of the cell to the center of the patch, and the angle of individual vectors relative to the mean vector of the field was calculated. Angles were color-coded according to the given scheme. (**b-d'**) Graphical representation of circular statistical analysis using Watson U^2 test (controls: 358 cells, *Lrp2*$^{-/267}$ mutants: 374 cells, 5 animals per genotype) documenting impaired coordination of ciliary patch displacement (VpatchD, $p<0.001$; b-b') as well as beating orientation (VpatchO, $p<0.05$; c-c') in receptor-deficient as compared to control cells. Also, alignment of patch displacement and beating orientation (VpatchD&O; d-d') is significantly reduced in mutants as shown by Watson U^2 test (controls: 337 cells, *Lrp2*$^{-/267}$: 342 cells, 5 animals per genotype; $p<0.001$).

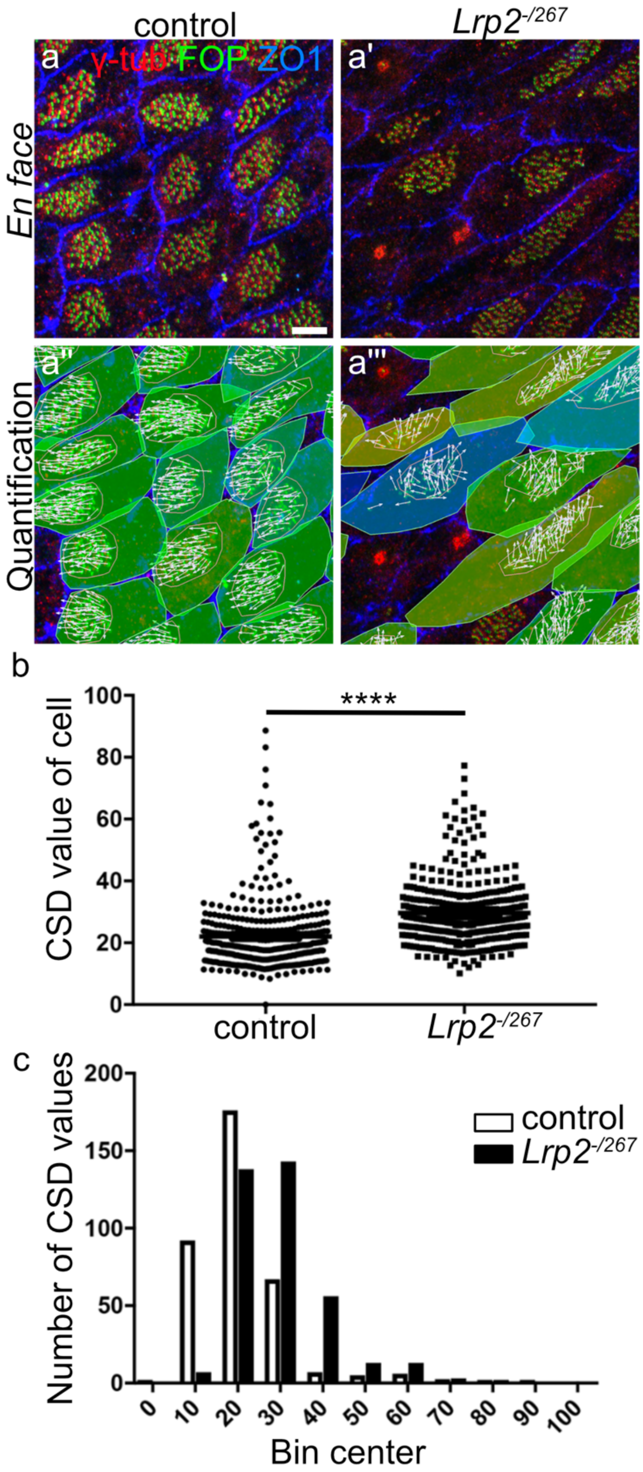

Supplementary Figure 3: Uncoordinated beating orientation of individual cilia within a ciliary patch of the LRP2-deficient adult ependyma

(**a-a'**) *En face* preparations of the LW of adult control and *Lrp2*$^{-/267}$ mice (P>70) stained for FOP (green), γ-tubulin (red), and ZO1 (blue). Scale bar: 5 µm. (**a''-a'''**) Panels depict the same images as in a-a' but visualizing individual cilia beating direction (direction of arrow)

projected on ependymal cells. Arrows indicate the vector from FOP to γ-tubulin signals and are defined by Vcil. (**b**) Vcils are used to determine the beating coordination of cilia in single cells given by circular standard deviation (CSD) value. CSD values are significantly lower in control (391 cells from 5 different animals, one dot per cell) as compared to *Lrp2*$^{-/267}$ mice (363 cells from 5 different animals; unpaired t test, $p<0.0001$). (**c**) Random beating orientation in mutant cells is also reflected by the frequency interval demonstrating more control cells (y-axis) with lower CSD value (x-axis) as compared to *Lrp2*$^{-/267}$ cells.

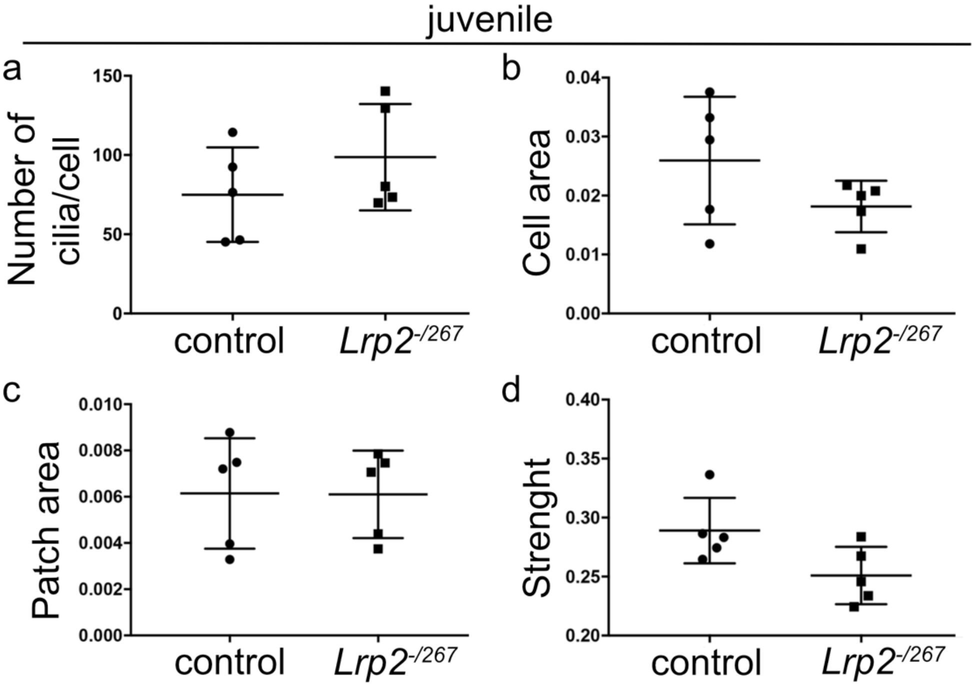

Supplementary Figure 4: Ciliary patch organization in ependymal cells of LRP2-deficient juvenile mice

Using Biotool1 software and analyzing 5 animals per genotype (265 cells in control and 284 cells in *Lrp2*$^{-/267}$ mice), no significant changes are detected in (**a**) the number of cilia per cell, in (**b**) cell surface area (Cell area), in (**c**) ciliary patch size (Patch area) and in (**d**) displacement of the ciliary patch relative to the cell center (Strenght) between the two analyzed genotypes.

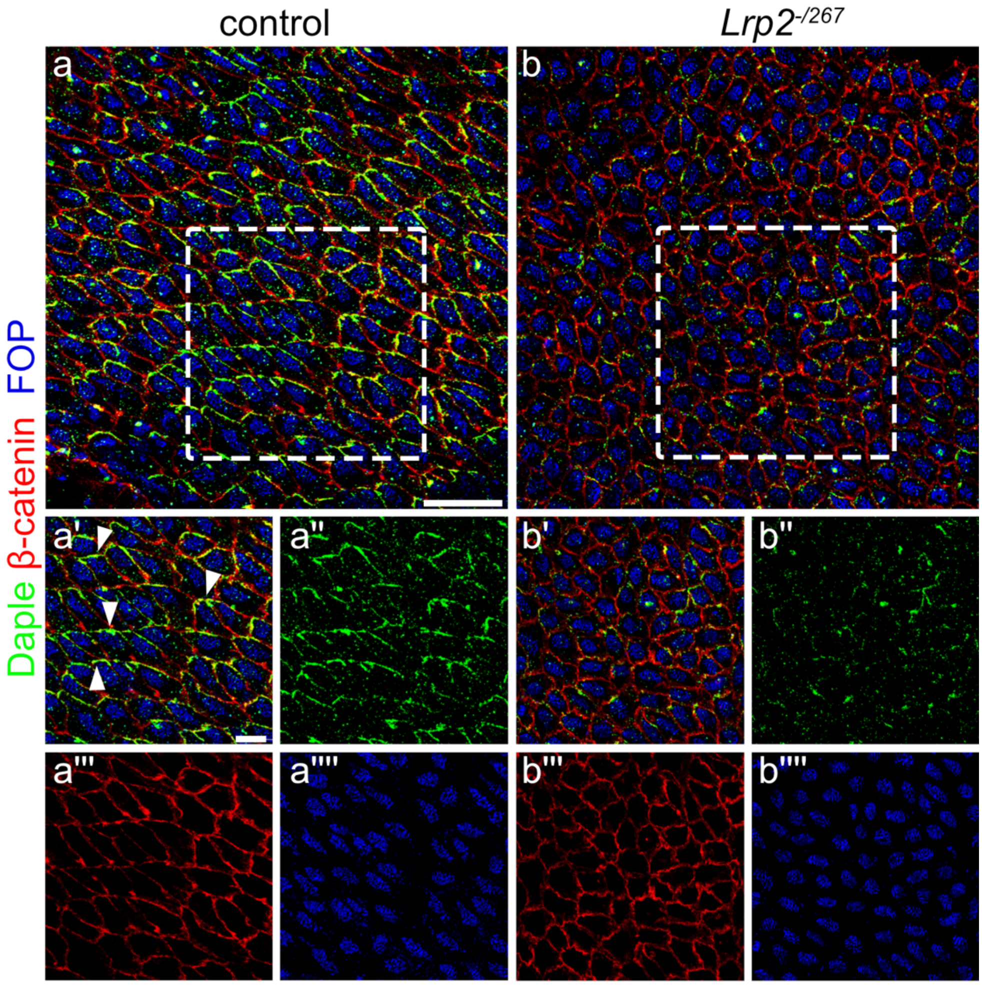

Supplementary Figure 5: Reduced and mislocalized Daple protein in the adult ependyma of LRP2-deficient mice

Whole mount immunodetection of LW preparations stained for Daple (green), β-catenin (red) and FOP (blue). (**a, b**) Merged overview pictures are shown for both genotypes. Scale bar: 25 µm. (**a-a'''', b-b''''**) Detailed comparison of Daple protein immunosignals in the indicated genotypes are given in single as well as merged channel configuration in higher magnification images. Polarized Daple distribution is detected in control mice (a', arrowheads). Significant protein reduction as well as lost coordinated protein distribution is demonstrated in *Lrp2*$^{-/267}$ mice (b' and b''). Scale bar: 10 µm.
